# Heme oxygenase-1 accelerates erastin-induced ferroptotic cell death

**DOI:** 10.18632/oncotarget.5162

**Published:** 2015-09-10

**Authors:** Min-Young Kwon, Eunhee Park, Seon-Jin Lee, Su Wol Chung

**Affiliations:** ^1^ School of Biological Sciences, College of Natural Sciences, University of Ulsan, Ulsan 680-749, South Korea; ^2^ Genome Structure Research Center, Korea Research Institute of Bioscience and Biotechnology, Yuseong-gu, Daejeon 305-806, South Korea

**Keywords:** heme oxygenase-1, oncology, oncogene, iron, free radicals

## Abstract

The oncogenic RAS-selective lethal small molecule Erastin triggers a unique iron-dependent form of nonapoptotic cell death termed ferroptosis. Ferroptosis is dependent upon the production of intracellular iron-dependent reactive oxygen species (ROS), but not other metals. However, key regulators remain unknown. The heme oxygenase (HO) is a major intracellular source of iron. In this study, the role of heme oxygenase in Erastin-triggered ferroptotic cancer cell death has been investigated. Zinc protoporphyrin IX (ZnPP), a HO-1 inhibitor, prevented Erastin-triggered ferroptotic cancer cell death. Furthermore, Erastin induced the protein and mRNA levels of HO-1 in HT-1080 fibrosarcoma cells. HO-1^+/+^ and HO-1^−/−^ fibroblast, HO-1 overexpression, and chycloheximide-treated experiments revealed that the expression of HO-1 has a decisive effects in Erastin-triggered cell death. Hemin and CO-releasing molecules (CORM) promote Erastin-induced ferroptotic cell death, not by biliverdin and bilirubin. In addition, hemin and CORM accelerate the HO-1 expression in the presence of Erastin and increase membranous lipid peroxidation. Thus, HO-1 is an essential enzyme for iron-dependent lipid peroxidation during ferroptotic cell death.

## INTRODUCTION

Cell death is crucial for normal development, homeostasis, and the prevention of hyperproliferative diseases such as cancer [1, 2]. The RAS family of small GTPases (HRAS, NRAS, and KRAS) is mutated in around 30% of all cancers [3]. Dolma et al and Yang et al identified two structurally unrelated small molecules, named Erastin and RSL3, that were selectively lethal to oncogenic RAS mutant cell lines and that they refer to together as RAS-selective lethal (RSL) compounds [4, 6]. The type of cell death activated by the RSLs has been enigmatic. Classic features of apoptosis, such as mitochondrial cytochrome c release, caspase activation, and chromatin fragmentation, are not observed in RSL-treated cells [4, 5, 6]. RSL-induced death is, however, associated with increased levels of intracellular reactive oxygen species (ROS) and is prevented by iron chelation or genetic inhibition of cellular iron uptake [5, 6]. In a recent systematic study of various mechanistically unique lethal compounds, the prevention of cell death by iron chelation was a rare phenomenon [7], suggesting that few triggers can access iron-dependent lethal mechanisms. They find that Erastin-induced death involves a unique constellation of morphological, biochemical, and genetic features, which led Stockwell and colleagues to propose the name ferroptosis as a description for this phenotype. Ferroptosis involves metabolic dysfunction that results in the production of both cytosolic and lipid ROS, independent of mitochondria but dependent on NADPH oxidases in some cell contexts [8].

Heme oxygenase-1 (HO-1) metabolizes heme to generate carbon monoxide (CO), biliverdin, and iron. Biliverdin is subsequently metabolized to bilirubin by biliverdin reductase [9, 10, 11]. HO-1 is contained that anti-cancer, anti-inflammatory, anti-apoptotic, anti-proliferative, and antioxidant effects [12, 13]. The expression of HO-1 has been shown to be up-regulated in different cancer type [14, 15, 16], but the role it plays in caners has not yet been addressed clearly. Especially, overexpressed or sustained HO-1 alters iron homeostasis in different cell types [17, 18]. In this study, we describe that Erastin induces HO-1 expression in HT-1080 fibrosarcoma cells and overexpressed HO-1 accelerates Erastin-triggered ferroptotic cell death.

## RESULTS

### Erastin induces heme oxygenase-1-dependent ferroptotic cell death

**Figure 1:**
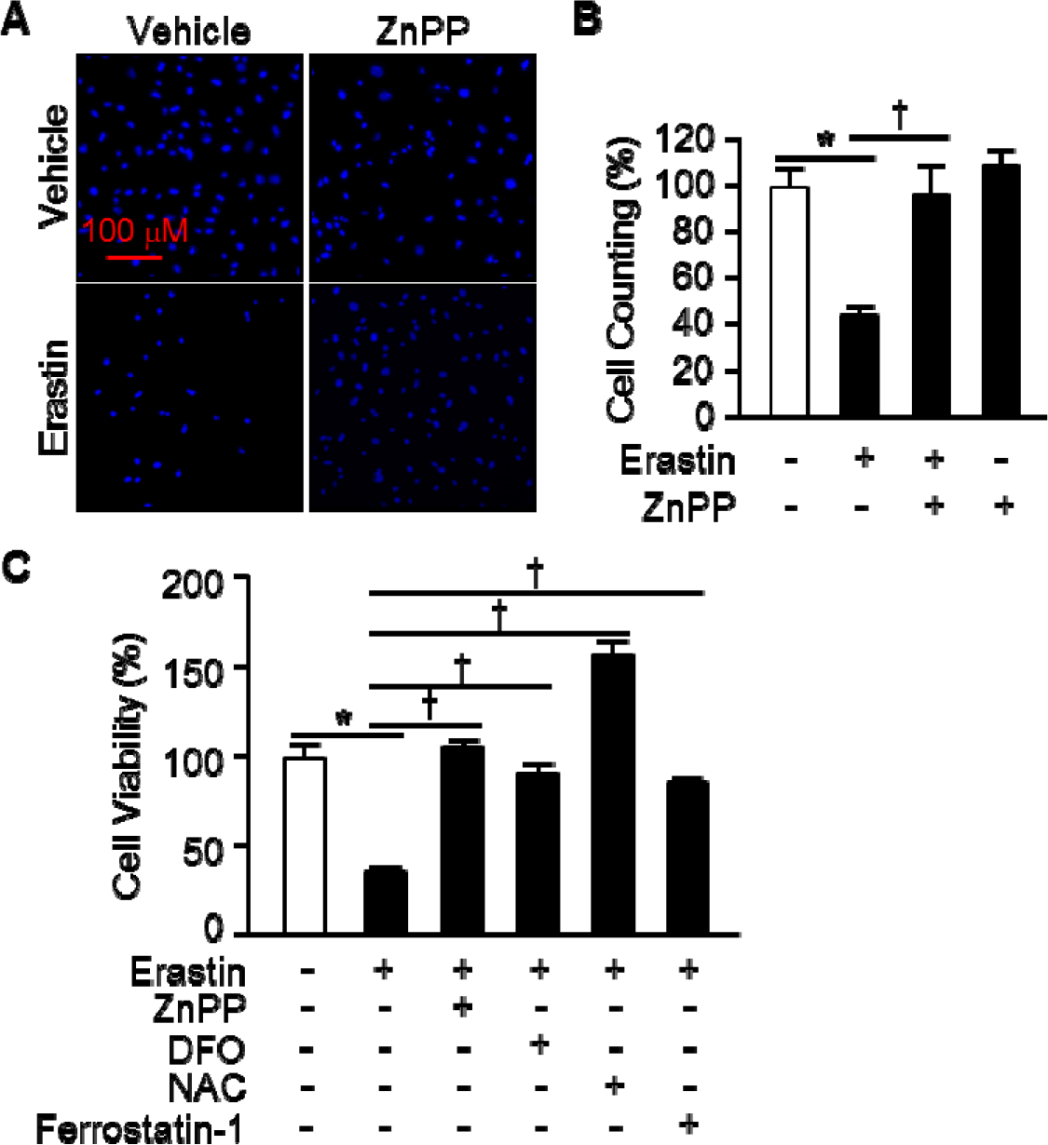
HO-1 inhibitor suppresses Erastin-induced ferroptotic death **A.** HT-1080 fibrosacoma cells were treated vehicle or Erastin (10 μM) in the presence or absence of ZnPP (zinc protoporphyrin, 10 μM). After 12 hours treatment, cell nucleus was stained to detect live cells using Hoechst 33342. **B.** The number of live cells was measured and represented as a graph. Values are mean ± SD, *n* = 7. *p < 0.05 *vs* vehicle, †*p* < 0.05 *vs* Erastin. **C.** Cell viability of HT-1080 fibrosacoma cells was measured at 12 hours after vehicle, Erastin (10 μM), or Erastin plus ZnPP, DFO (deferoxamine, 100 μM), NAC (N-acetylcysteine, 10 mM), or ferrostatin-1 (0.5 μM) administration using Ez-Cytox Cell Viability Assay Kit. *p < 0.05 *vs* vehicle, †*p* < 0.05 *vs* Erastin. Values are mean ± SD, *n* = 12.

The oncogenic RAS-selective lethal small molecule Erastin triggers a unique iron-dependent form of nonapoptotic cell death, ferroptosis [19]. A link between heme oxygenase (HO) and iron homeostasis has been demonstrated in different tissues and cells [20, 21]. To investigate the effects of HO-1 inhibitor, zinc protoporphyrin (ZnPP), we treated HT-1080 fibrosarcoma cells with vehicle or Erastin (10 μM) in the absence or presence of ZnPP (10 μM) for 12 hours. The cells were harvested and stained for nuclei using Hoechst 33342. The representative images were shown in Figure 1A. Live cells were counted and represented as a graph in Figure 1B. HO-1 inhibitor, ZnPP, prevented Erastin-induced cell death. To verify these results, we assessed Erastin-induced cell death in the absence or presence of ZnPP, N-acetylcysteine (NAC, antioxidant), ferrostatin-1 (ferroptosis inhibitor), or deferoxamine (DFO, iron chelator) as a control. The cell viability of HT-1080 fibrosarcoma cells was decreased in Erastin (33.9%) compared with vehicle (100%). However, the Erastin-induced cell death was obstructed by HO-1 inhibitor, ZnPP (104.1%), DFO (87.5%), NAC (154.1%), and ferrostatin-1 (85%) (Figure 1C). These data suggest that HO-1 may have a key role in the Erastin-triggered iron-dependent cell death, ferroptosis.

### The expression of heme oxygenase-1 is an important for Erastin-induced ferroptotic cell death

**Figure 2:**
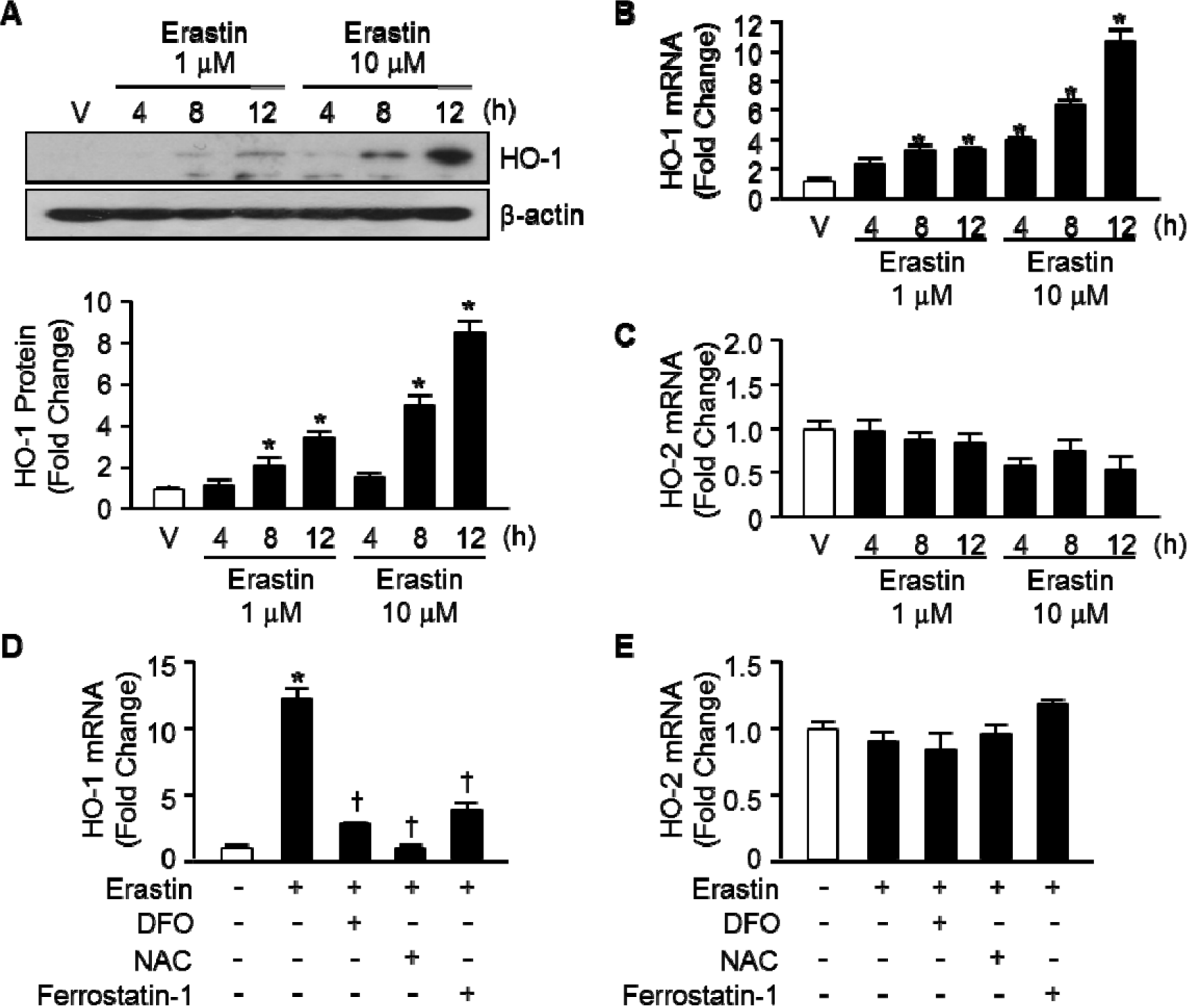
Erastin induces HO-1 expression in HT-1080 fibrosacoma cells **A.** Western blotting for HO-1 was performed 4, 8, and 12 hours after vehicle (V), or Erastin (1 μMor 10 μM) in HT-1080 fibrosacoma cells. β-actin was used as controls for normalization. This represents a representative blot of three independent experiments. The fold change in protein levels were quantitated as signal intensity corrected for loading in vehicle or Erastin treated cells. *p < 0.05 *vs* vehicle. Values are mean ± SD, *n* = 3. Quantitative real-time PCR was performed to assess mRNA levels of HO-1 **B.** and HO-2 **C.** in HT-1080 fibrosacoma cells. *p < 0.05 *vs* vehicle. Quantitative real-time PCR was performed to assess mRNA levels of HO-1 **D.** and HO-2 **E.** 12 hours after vehicle, Erastin, or Erastin plus DFO, NAC, or ferrostatin-1 administration in HT-1080 fibrosacoma cells. *p < 0.05 *vs* vehicle, †*p* < 0.05 *vs* Erastin. Values are mean ± SD, *n* = 3.

To determine Erastin affects the expression levels of HO-1, we treated HT-1080 fibrosarcoma cells with vehicle or Erastin (1 μM or 10 μM) and harvested total protein and RNA at 4, 8, and 12 hours after cell treatment. HO-1 protein and mRNA levels began to increase 8 hours and a more striking increase in HO-1 was evident after 12 hours of Erastin treatment (Figure 2A, and 2B, respectively). Furthermore, Erastin increased the protein and mRNA levels of HO-1 dose dependently. However, the expression levels of HO-2 did not alter during Erastin-triggered cell death (Figure 2C). In Figure 1C, Erastin-induced cell death was recovered in the presence of DFO, NAC, and ferrostatin-1 in HT-1080 fibrosacoma cells. To investigate the role of HO-1 in the condition, the expression levels of HO-1 were assessed 12 hours after vehicle, Erastin, or Erastin plus DFO, NAC, or ferrostatin-1 administration in HT-1080 fibrosacoma cells. Whereas the expression levels of HO-1 were increased by Erastin, it was down-regulated by DFO, NAC, and ferrostatin-1 in the presence of Erastin in HT-1080 fibrosacoma cells (Figure 2D). However, the expression levels of HO-2 did not change in the same condition (Figure 2E).

**Figure 3:**
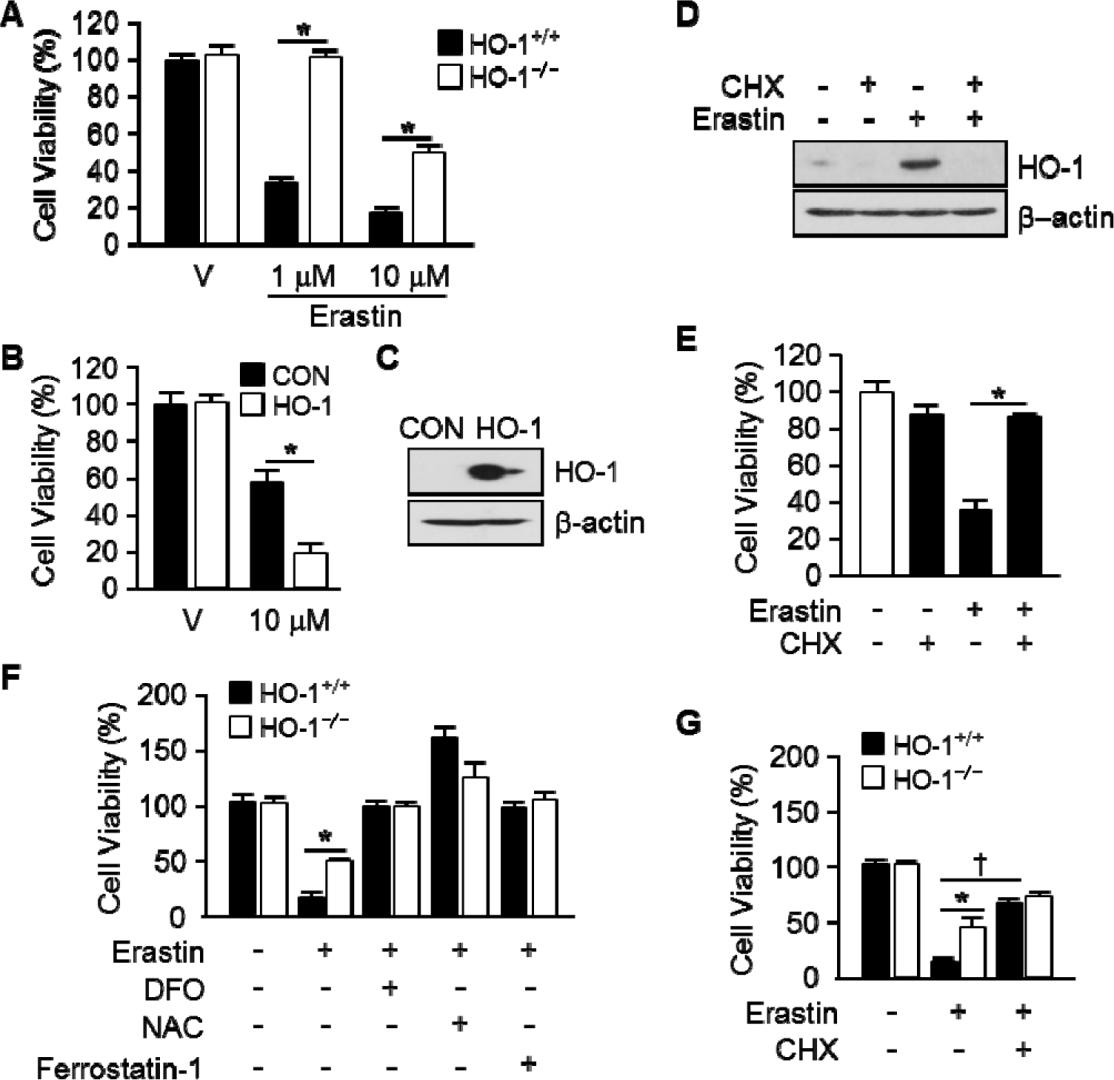
The expression of HO-1 is an important for Erastin-induced ferroptotic cell death **A.** Cell viability was measured 12 hours after vehicle or Erastin (1 μMor 10 μM) administration in mouse HO-1^+/+^ and HO-1^−/−^ fibroblasts using Ez-Cytox Cell Viability Assay Kit. *p < 0.05 HO-1^−/−^ *vs* HO-1^+/+^. Values are mean ± SD, *n* = 12. **B.** HT-1080 fibrosacoma cells were stably transfected with control vector (CON) or human HO-1 cDNA (HO-1). These groups of cells were exposed to vehicle or Erastin (10 μM). Cell viability was measured 12 hours after treatment in control vector (CON) or human HO-1 overexpressed HT-1080 fibrosacoma cells using Ez-Cytox Cell viability Assay Kit. *p < 0.05 HO-1 overexpressed *vs* control vector expressed HT-1080 fibrosacoma cells in the presence of Erastin. Values are mean ± SD, *n* = 12. **C.** The overexpression of human HO-1 was detected using western blotting for HO-1. **D.** CHX was treated in HT-1080 fibrosacoma cells for 12 hours in the absence or presence of Erastin. The expression of HO-1 was detected using Western blotting analysis and β-actin was used for loading control. **E.** Cell viability was measured 12 hours after vehicle or Erastin (10 μM) administration in the absence or presence of CHX. *p < 0.05 *vs* Erastin. Values are mean ± SD, *n* = 9. **F.** Cell viability was measured 12 hours after vehicle, Erastin (10 μM), or Erastin plus DFO, NAC, ferrostatin-1 administration in mouse HO-1^+/+^ and HO-1^−/−^ fibroblasts using Ez-Cytox Cell Viability Assay Kit. *p < 0.05 HO-1^−/−^ *vs* HO-1^+/+^. Values are mean ± SD, *n* = 11. **G.** Cell viability was measured 12 hours after vehicle, Erastin (10 μM), or Erastin plus CHX in mouse HO-1^+/+^ and HO-1^−/−^ fibroblasts using Ez-Cytox Cell Viability Assay Kit. *p < 0.05 HO-1^−/−^ *vs* HO-1^+/+^, †*p* < 0.05 Erastin plus CHX *vs* Erastin. Values are mean ± SD, *n* = 12.

To ascertain the importance of HO-1 expression in Erastin-triggered cell death, we harvested the lung fibroblastic cells from HO-1^+/+^ and HO-1^−/−^ mice. Vehicle or Erastin (1 μM, 10 μM) was treated in HO-1^+/+^ or HO-1^−/−^ fibroblastic cells for 12 hours and cell viability was measured. The cell death of HO-1^−/−^ fibroblastic cells was prevented in Erastin 1 μM (102.3%) or 10 μM (49.6%) compared with Erastin 1 μM (32.4%) or 10 μM (17.6%) treatment in HO-1^+/+^ fibroblastic cells (Figure 3A). In addition, HT-1080 fibrosarcoma cells were transfected with control vector or human HO-1 cDNA to see the effects of overexpressed HO-1 in Erastin-induced cell death. Transfected cells were treated with vehicle or Erastin (10 μM) for 12 hours and cell viability was measured. The Erastin-induced cell death was accelerated in HO-1 overexpressed HT-1080 fibrosarcoma cells in the presence of Erastin 10 μM (19.0%) compared with Erastin 10 μM (57.3%) treatment in control vector overexpressed HT-1080 fibrosarcoma cells (Figure 3B). The overexpression of control vector or human HO-1 cDNA was detected using anti-HO-1 antibody at the baseline (Figure 3C). To verify the importance of HO-1 expression, an inhibitor of protein biosynthesis, cycloheximide (CHX), was treated in HT-1080 fibrosarcoma cells in the absence or presence of Erastin. The protein levels of HO-1 were determined using Western blotting with or without Erastin in the absence or presence of CHX (Figure 3D). As we expected, CHX prevented the expression of Erastin-induced HO-1 protein. In the same condition, the cell viability was measured. The cell viability of HT-1080 fibrosarcoma cells was decreased in Erastin (35.1%) compared with vehicle (100%). However, Erastin-induced cell death was prevented by an inhibitor of protein biosynthesis (85.9%), CHX (Figure 3E). We also investigated whether the expression of HO-1 affects the cell viability after vehicle, Erastin, or Erastin plus DFO, NAC, ferrostatin-1, or CHX administration in HO-1^+/+^ or HO-1^−/−^ fibroblastic cells. DFO, NAC, and ferrostatin-1 rescued Erastin-induced cell death in HO-1^+/+^ and HO-1^−/−^ fibroblastic cells (Figure 3F). Interestingly, CHX could not rescue Erastin-induced cell death in HO-1^−/−^ fibroblastic cells compare to HO-1^+/+^ fibroblastic cells (Figure 3G). These data suggest that the expression of HO-1 is an important mediator of Erastin-triggered iron-dependent cell death, ferroptosis.

### By products of heme oxygenase-1 accelerate the Erastin-induced ferroptotic cell death

**Figure 4:**
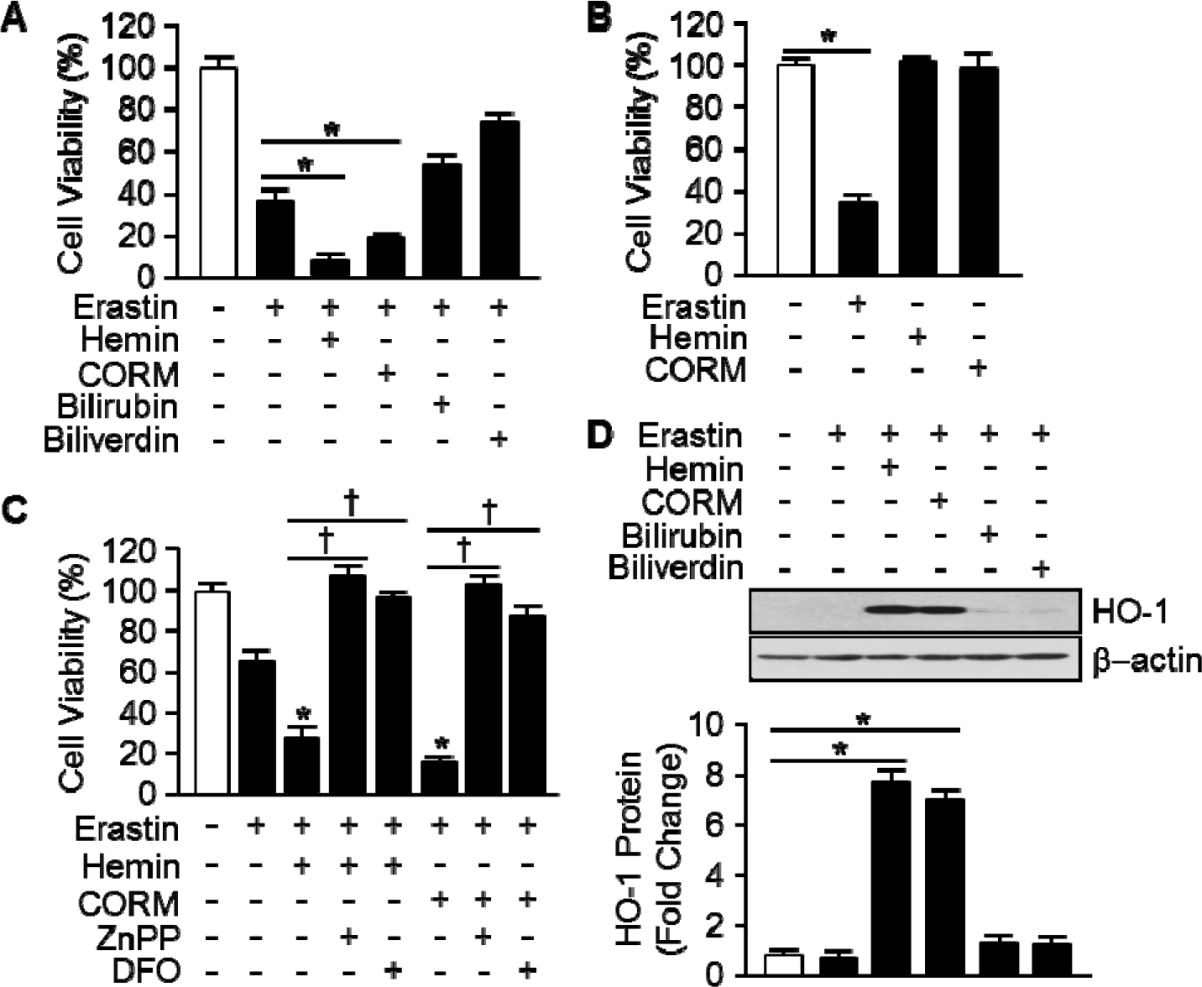
Hemin and CORM accelerate Erasitn-induced ferroptotic cell death in HT-1080 fibrosacoma cells **A.** Cell viability was analyzed 8 hours after vehicle, Erastin (10 μM), and hemin (5 μM), CORM (10 μM), bilirubin (10 μM), or biliverdin (10 μM)plus Erastin (10 μM)administration in HT-1080 fibrosacoma cells using Ez-Cytox Cell viability Assay Kit. *p < 0.05 *vs* Erastin. Values are mean ± SD, *n* = 8. **B.** Cell viability was analyzed 8 hours after vehicle, Erastin (10 μM), and hemin (5 μM) or CORM (10 μM)administration in HT-1080 fibrosacoma cells using Ez-Cytox Cell viability Assay Kit. *p < 0.05 *vs* vehicle. Values are mean ± SD, *n* = 8. **C.** Cell viability was analyzed 8 hours after vehicle, Erastin (10 μM), or Erastin plus hemin (5 μM)or CORM (10 μM) in the absence or presence of ZnPP or DFOadministration in HT-1080 fibrosacoma cells using Ez-Cytox Cell viability Assay Kit. *p < 0.05 *vs* Erastin, †*p* < 0.05 *vs* Erastin plus hemin or CORM. Values are mean ± SD, *n* = 8. **D.** Total protein was harvested 8 hours after vehicle, Erastin, or Erastin plus hemin (5 μM), CORM (10 μM), bilirubin (10 μM), or biliverdin (10 μM) administration in HT-1080 fibrosacoma cells. Western blotting for HO-1 was performed and β-actin was used as controls for normalization. This represents a representative blot of three independent experiments. The fold change in protein levels were quantitated as signal intensity corrected for loading in vehicle or Erastin treated cells. *p < 0.05 *vs* vehicle. Values are mean ± SD, *n* = 3.

**Figure 5:**
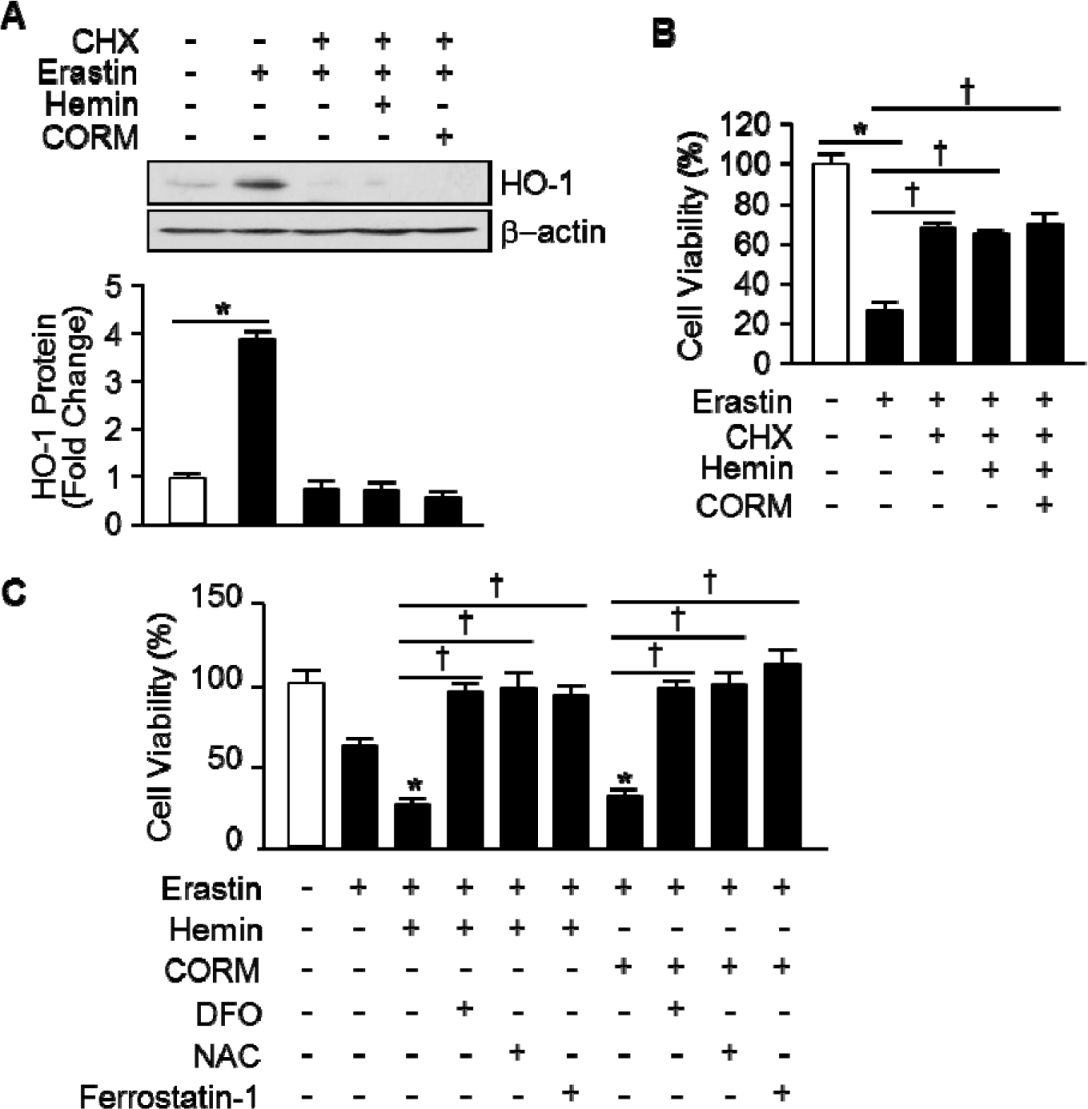
The effects of hemin and CORM on Erastin-induced ferroptotic cell death play through the induction of HO-1 expression **A.** Total protein was harvested 8 hours after vehicle, Erastin (10 μM), and hemin (5 μM) or CORM (10 μM) plus Erastin administration in the absence or presence of CHX (1 μg/mL) in HT-1080 fibrosacoma cells. Western blotting for HO-1 was performed and β-actin was used as controls for normalization. This represents a representative blot of three independent experiments. The fold change in protein levels were quantitated as signal intensity corrected for loading in vehicle or Erastin treated cells. *p < 0.05 *vs* vehicle. Values are mean ± SD, *n* = 3. **B.** Cell viability was analyzed 8 hours after vehicle, Erastin (10 μM), or Erastin plus hemin (5 μM) or CORM (10 μM)administration in the absence or presence of CHX in HT-1080 cells. *p < 0.05 vehicle, †*p* < 0.05 *vs* Erastin. Values are mean ± SD, *n* = 8. **C.** Cell viability was analyzed 8 hours after vehicle, Erastin (10 μM), and Erastin plus hemin (5 μM) or CORM (10 μM)administration in the absence or presence of DFO, NAC, or ferrostatin-1 in HT-1080 fibrosacoma cells. *p < 0.05 *vs* Erastin, †*p* < 0.05 *vs* Erastin plus hemin or CORM. Values are mean ± SD, *n* = 12.

To ascertain by products of HO-1 promote Erastin-induced ferroptotic cell death, hemin, CORM, biliverdin, and bilirubin were treated and cell viability was measured. Erastin plus hemin or CORM treated cells were started to die much earlier (6 hours) than Erastin alone (10 hours). However, biliverdin and bilirubin had no effects on Erastin-induced cell death (Figure 4A). The effects of hemin or CORM were suppressed by cotreatment with iron chelator, deferoxamine (DFO, 100 μM) or HO-1 enzyme inhibitor, ZnPP (10 μM) (Figure 4C). In the other hands, hemin or CORM does not induce cell death, alone (Figure 4B). Interestingly, Hemin and CORM increased HO-1 expression much earlier at 6 hours in the presence of Erastin (Figure 4D). At the time, Erastin showed basal expression of HO-1. The fold change in HO-1 protein levels were quantitated as signal intensity corrected for loading in control cells in Figure 4D. To verify the importance of HO-1 expression, cells were treated with vehicle, Erastin, or Erastin plus hemin or CORM in the absence or presence of cycloheximide (CHX) and protein levels of HO-1 were analyzed by Western blotting analysis (Figure 5A). CHX prevents the protein levels of HO-1 in the presence of Erastin or Erastin plus hemin or CORM. The fold change in HO-1 protein levels were quantitated as signal intensity corrected for loading in control cells in Figure 5A on the bottom. In Figure 5B, data shown that the obstruction of HO-1 expression prevents Erastin or Erastin plus hemin or CORM-induced cell death in the presence of CHX. HT-1080 fibrosacoma cells were treated with vehicle, Erastin, or Erastin plus hemin or CORM in the presence of DFO, NAC, or ferrostatin-1 and cell viability was analyzed. The accelerated cell death by hemin and CORM was rescued by DFO, NAC, and ferrostatin-1 in the presence of Erastin (Figure 5C). These data suggest that by products of heme oxygenase-1, hemin and CORM, accelerate the Erastin-induced ferroptotic cell death in HT-1080 fibrosacoma cells.

### Heme oxygenase-1 induces lipid peroxidation in Erastin-treated cells

**Figure 6:**
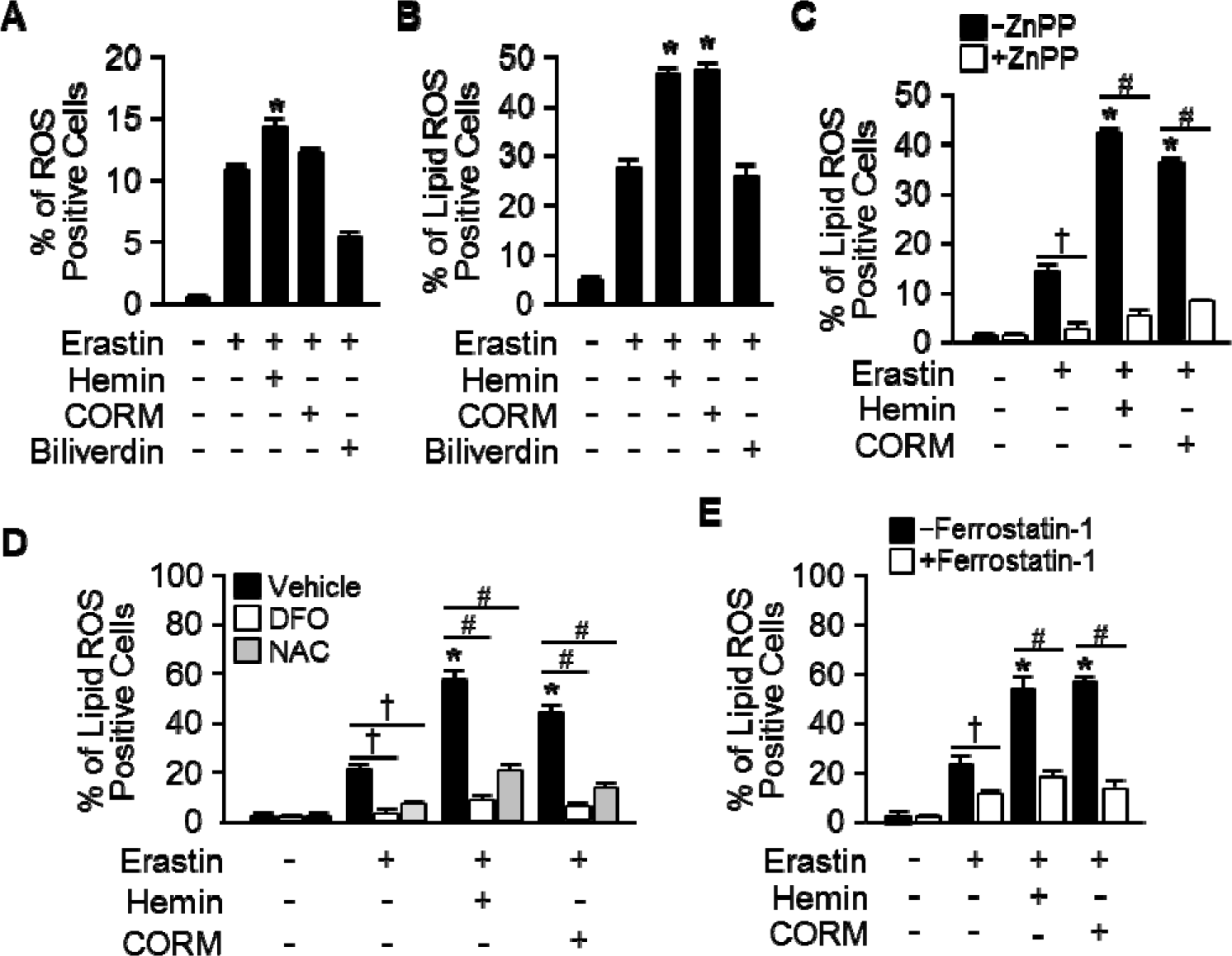
Hemin and CORM accelerate the accumulation of lipid peroxidation in the presence of Erastin HT-1080 fibrosacoma cells were treated with vehicle, Erastin (10 μM), or hemin (5 μM), CORM (10 μM), or biliverdin (10 μM) in the presence of Erastin. Cytosolic ROS **A.** and lipid peroxidation **B.** were assayed by flow cytometry using the fluorescent probes CellROX^®^ Deep Red (cytosolic ROS) and C11-BODIPY (lipid peroxidation), respectively. Values are mean ± SD, *n* = 6. *p < 0.05 *vs* Erastin. **C.** HT-1080 fibrosacoma cells were treated vehicle, Erastin, Erastin plus hemin, or CORM in the presence or absence of HO-1 inhibitor, ZnPP (zinc protoporphyrin, 10 μM). Lipid peroxidation was analyzed. Values are mean ± SD, *n* = 6. *p < 0.05 *vs* Erastin, †*p* < 0.05 *vs* Erastin, #*p* < 0.05 *vs* Erastin plus hemin or CORM. **D.** Lipid peroxidation was analyzed after vehicle (0.1% DMSO), Erastin, or Erastin plus hemin or CORM administration in the absence or presence of DFO or NAC in HT-1080 fibrosacoma cells. Values are mean ± SD, *n* = 9. *p < 0.05 *vs* Erastin, †*p* < 0.05 *vs* Erastin, #*p* < 0.05 *vs* Erastin plus hemin or CORM. **E.** Lipid peroxidation was analyzed after vehicle (0.1% DMSO), Erastin, or Erastin plus hemin or CORM administration in the absence or presence of ferrostatin-1 in HT-1080 fibrosacoma cells. Values are mean ± SD, *n* = 9. *p < 0.05 *vs* Erastin, †*p* < 0.05 *vs* Erastin, #*p* < 0.05 *vs* Erastin plus hemin or CORM.

Erastin-induced cell death is a poorly characterized process involving the accumulation of ROS derived from an unknown source and the inhibition of cell death by iron chelation [5, 6]. To investigate the effects of HO-1 in lipid peroxidation, HT-1080 fibrosacoma cells were treated with vehicle, Erastin, or hemin, CORM, and biliverdin in the presence of Erastin. Cytosolic reactive oxygen species (ROS) (Figure 6A) and lipid peroxidation (Figure 6B) were assayed by flow cytometry using the fluorescent probes CellROX and C11-BODIPY, respectively. As shown in Figure 6A, hemin increased Erastin-induced cytosolic ROS, but CORM did not. Even, biliverdin decreased Erastin-induced cytosolic ROS. Interestingly, hemin and CORM increased lipid peroxidation in the presence of Erastin in Figure 6B. These data suggest that hemin and CORM accelerate Erastin-triggered cell death through increasing lipid peroxidation. In Figure 1C, HO-1 inhibitor, ZnPP, prevented Erastin-induced cell death. To investigate the role of HO-1 during lipid peroxidation, vehicle, Erastin, or Erastin plus hemin or CORM was treated in the presence or absence of HO-1 inhibitor, ZnPP and lipid peroxidation was analyzed in Figure 6C. In addition, hemin or CORM increased lipid peroxidation in the absence of ZnPP. However, hemin and CORM-induced lipid peroxidation were abolished in the presence of ZnPP. Lipid peroxidation of Eratsin was reduced in the presence of ZnPP in Figure 6C. In Figure 5C, DFO, NAC, and ferrostain-1 prevented hemin and CORM-induced cell death in the presence of Erastin. We wondered that their inhibition effects were going through the inhibition of lipid peroxidation. For this reason, lipid peroxidation was analyzed after vehicle, Erastin, or Erastin plus hemin or CORM in the absence or presence of DFO, NAC, or ferrostatin-1 (Figure 6D and 6E). DFO, NAC, and ferrostatin-1 decreased Erastin plus hemin or CORM-induced lipid peroxidation. These data suggest that HO-1 may be an important enzyme in Erastin-triggered cell death through increasing lipid peroxidation.

## DISCUSSION

Yang WS and his colleagues reported the identification of additional small molecules, RSL3, ML162, and DPI10 [5, 22], that display oncogenic-RAS-synthetic-lethality in engineered fibroblast-derived tumorigenic cell lines. Initially, they focused on Erastin. Erastin reprograms cancer cell metabolism by modulating VDAC2/VDAC3 and system xc- to trigger ferroptosis [6, 8]. Ferroptotic cell death is morphologically, biochemically, and genetically distinct from apoptosis, various forms of necrosis, and autophagy. This process is characterized by the overwhelming, iron-dependent accumulation of lethal lipid ROS [8]. Unlike other forms of apoptotic and nonapoptotic death [23, 24], this requirement for ROS accumulation appears to be universal. In at least some cells, NOX family enzymes make important contributions to this process. Indeed, although Dixon and his colleagues cannot exclude the possibility of a death-inducing protein or protein complex activated downstream of ROS accumulation, they posit that the executioners of death in cancer cells undergoing ferroptosis are these ROS themselves. However, RSL-induced death is prevented by iron chelation or genetic inhibition of cellular iron uptake. This means that the regulation of intracellular iron content is a key factor to induce ferroptosis. Heme oxygenase-1 initiates the catabolism of heme, releasing carbon monoxide, iron, and biliverdin. So, HO-1 could involve in the process of RSL-induced ferroptotic cell death. As Figure 1 shown, HO-1 inhibitor, ZnPP, prevents Erastin-induced ferroptotic cell death, completely. Also, the protein and mRNA levels were increased by Erastin itself. These data suggest that enzymatic functions and expression of HO-1 may important for Erastin-induced cell death, ferroptosis. This hypothesis supported by the data from Figure 3. The effects of cancer cell death were decreased in HO-1 null fibroblast and overexpression of HO-1 accelerated the cancer cell death in the presence of Erastin. In Figure 3D and 3E, the translational inhibition experiments of HO-1 revealed the importance of the expression of HO-1. Once more, Figure 4 and 5 imply the importance of HO-1 expression in Erastin-induced ferroptotic cell death. HO-1 is a well-known antioxidant enzyme [25]. Moreover, antioxidant effects of hemin and CORM are published in different conditions [26, 27], also. Their antioxidant effects contribute the inhibition of cancer cells, such as breast and renal cancer cells [28, 29, 30]. However, hemin and CORM increased the lipid peroxidation in the presence of Erastin in Figure 6. This might be through increase of intracellular iron contents. Taken together, HO-1 may take part in iron supplement and lipid peroxidation in Erastin-induced ferropotic cell death.

## MATERIALS AND METHODS

### Cell culture

HT-1080 fibrosacoma cells were grown in RPMI 1640 medium (Life Technologies, Grand Island, NY) supplemented with 10% fetal bovine serum (Invitrogen, Life technologies, Carlsbad, CA), penicillin (100 u/mL), and streptomycin (100 μg/mL). All cells were incubated at 37°C in a humidified atmosphere of 5% CO_2_ and 95% air.

### Fibroblast isolation and culture

Mouse lung fibroblasts were isolated from HO-1^+/+^ or HO-1^−/−^ mice (Balb/c background). For isolation of Fibroblast, lungs were perfused with 20 mL of Hank's Balanced Salt Solution (HBSS), minced with scissors and finally digested for 30 minutes with collagenase type I (Worthington, Lakewood, NJ) at 37°C. Digested extracts were pressed through 70 μm cell strainers. The filtrate was centrifuged at 1500 rpm for 5 minutes at 25°C. After remove the supernatant, pellets were resuspended in DMEM supplemented with 10% FBS, 100 U/mL penicillin, 100 μg/mL streptomycin, Fungizone^®^ Antimycotic (Invitrogen, Life technologies, Carlsbad, CA) 0.25 μg/mL. The cells were then seeded onto culture plates.

### Reagents and antibodies

Erastin (EMD Millipore Corporation, USA), Znpp-IX (ENZO Life Science, Plymouth Meeting, PA), Carbon monoxide-releasing molecule (CORM) (Sigma-Aldrich, St Louis, MO), hemin, bilirubin (Frontier Scientific, Inc, USA), and biliverdin (MP Biomedicals, LLC, France) were used. Deferoxamine (DFO) and cycloheximide were purchased from Sigma-Aldrich (St. Louis, MO). Anti- HO-1 was purchased from StressGen Biotechnologies Inc. (Victoria, BC, Canada). Anti-b-actin was purchased from Sigma-Aldrich (St Louis, MO). Horseradish peroxidase (HRP) conjugated goat anti-rabbit or goat anti-mouse IgG was from Santa Cruz Biotechnology, Inc. (Dallas, TX).

### Western immunoblotting

Cell extracts from the 60 mm dishes were harvested using RIPA buffer (Tris/Cl (pH 7.6); 100 mmole/L, EDTA; 5 mmole/L, NaCl; 50 mmole/L, β-glycerophosphate; 50 mmole/L, NaF; 50 mmole/L, Na_3_VO_4_; 0.1 mmole/L, NP-40; 0.5%, Sodium deoxycholate; 0.5%) with 1× Complete™ protease inhibitor Cocktail (Roche Applied Science, Mannheim, Germany). Protein concentrations of cell lysates were determined using Pierce BCA protein assay kit (Thermo Scientific, Rockford, IL) and were resolved by 12% SDS-polyacrylamide gels. Proteins were transferred on Pure PVDF membranes. Membranes were blocked for 2 hours at room temperature with a 5% nonfat milk solution in TBST buffer (20 mM Tris–HCl, pH 7.4, 500 mM NaCl, 0.1% Tween20). The blots were then incubated with antibody an anti-HO-1 (1:4000), an anti-β-actin (1:5000) in TBST overnight at room temperature. The blots were then washed three times in TBST and incubated with an anti-rabbit secondary antibody (1:5000), or an anti-mouse secondary antibody (1:5000) in TBST for 1 hour at room temperature. Finally, immunoblots were detected by SuperSignal^®^ West Pico Chemiluminescent Substrate (Thermo Scientific, Rockford, IL) and visualized after exposure to X-ray film.

### Quantitative real-time RT-PCR

Total RNA was isolated TRIzol reagent (Invitrogen, Life technologies, Carlsbad, CA), Reverse transcription was performed using SuperScript™ III First-Strand Synthesis System (Invitrogen, CA). Real-time quantitative PCR was conducted using iQ SYBR Green Supermix (Bio-Rad, Hercules, CA). Triplicate samples per condition were analyzed on an Applied Biosystems StepOnePlus^TM^ Real-Time PCR System using absolute quantification settings. The primers sequences were as follows: mouse HO-1 (forward: 5′-CGCCTTCCTGCTCAACATT-3′ and reverse: 5′-TGTGTTCCTCTGTCAGCATCAC-3′) mouse β-actin (forward: 5′-GATCTGGCACCACACCTTCT-3′ and reverse: 5′-GGGGTGTTGAAGGTCTCAAA-3′). Amplification of cDNA started with 10 minutes at 95°C, followed by 40 cycles of 15 seconds at 95°C and 1 minute at 60°C.

### Cell viability assay

Cell viability was determined by the MTS assay using the CellTiter 96^®^ AQueous One Solution Cell Proliferation Assay kit (Promega, Madison, WI). Cells were seeded at 0.7 × 10^4^ cells per well in 96-well plates. After reagent treatment, 20 μl of MTS solution was added to each well. Plates were incubated for an additional 2~4 hours at 37°C. Absorbance at 490 nm was then measured using a SpectraMax M2 microplate reader (Molecular Devices, Sunnyvale, CA) to calculate the cell survival percentages.

### Nuclear staining

Cells were seeded on 12-well plates containing glass coverslips. Next day, cells were treated with Erastin (1 μM, 10 μM) for 10 hours. In order to inhibit HO-1, ZnPP (10 μM) was pretreated for 30 minutes before the addition of Erastin. A nuclear counterstaining was made with a solution of 1 μg/mL Hoechst 33258 staining for 5 minutes and mounting on a slide using Fluorescence Mounting Medium (Dako, Glostrup, Denmark). The samples were visualized by Nikon Image Ti fluorescence microscope to acquire fluorescent images using NIS-Elements Br software.

Cell viability was assessed by fluorescence microscope in response to Erastin with ZnPP.

### HO-1 overexpression stable cell lines

The stable cell lines for HO-1 overexpression were generated in HT-1080 fibrosacoma cells. Cells were seeded in 6-well dishes the day before the experiment in regular HT-1080 media. The next day, pEGFP-N3 or pEGFP-N3-hHO-1 was transfected using FuGENE6 transfection reagent (Roche, Indianapolis, IN, USA) according to the manufacturer's instructions. 48 hours post-transfection, transfected cells were treated with neomycin-containing media (500 ng/mL for HT-1080) to obtain transfected cell populations. HO-1 overexpression was validated by western blotting analyses.

### Assessment of cytosolic ROS and lipid peroxidation

Cells were seeded at 3 × 10^5^ cells per well in 6-well plates. Next day, cells were treated with Erastin (10 μM) and/or hemin (5 μM), CORM (10 μM), biliverdin (10 μM) for 8 hours. After 8 hours, cells were incubated with 2 μM CellROX^®^ Deep Red (cytosolic ROS) or 2 μM C11-BODIPY581/591 (lipid peroxidation) (Invitrogen, Life Technologies, Grand Island, NY) for 30 minutes at 37°C in the dark. After 30 minutes of loading, unincorporated dye was removed by washings with 2% FBS containing PBS. Samples were then centrifuged at 1000 rpm for 3 minutes and the pellets were resuspended in 500 μL of 2% FBS containing PBS. Measurements were performed on a FACSCalibur (Becton Dickinson, San Jose, CA) flow cytometer.

### Statistical analysis

Data represent mean ± SD. For comparisons between two groups, we used Student's two-tailed unpaired *t* test. Statistically significant differences were accepted at *p* < 0.05.

## References

[1] Fuchs Y, Steller H (2011). Programmed cell death in animal development and disease. Cell.

[2] Thompson CB (1995). Apoptosis in the pathogenesis and treatment of disease. Science.

[3] Vigil D, Cherfils J, Rossman KL, Der CJ (2010). Ras superfamily GEFs and GAPs: validated and tractable targets for cancer therapy?. Nat. Rev. Cancer.

[4] Dolma S, Lessnick SL, Hahn WC, Stockwell BR (2003). Identification of genotype-selective antitumor agents using synthetic lethal chemical screening in engineered human tumor cells. Cancer Cell.

[5] Yang WS, Stockwell BR (2008). Synthetic lethal screening identifies compounds activating iron-dependent, nonapoptotic cell death in oncogenic-RAS-harboring cancer cells. Chem Biol.

[6] Yagoda N, von Rechenberg M, Zaganjor E, Bauer AJ, Yang WS, Fridman DJ, Wolpaw AJ, Smukste I, Peltier JM, Boniface JJ (2007). RAS-RAF-MEK-dependent oxidative cell death involving voltagedependent anion channels. Nature.

[7] Wolpaw AJ, Shimada K, Skouta R, Welsch ME, Akavia UD, Pe'er D, Shaik F, Bulinski JC, Stockwell BR (2011). Modulatory profiling identifies mechanisms of small molecule-induced cell death. Proc Natl Acad Sci USA.

[8] Dixon SJ, Lemberg KM, Lamprecht MR, Skouta R, Zaitsev EM, Gleason CE, Patel DN, Bauer AJ, Cantley AM, Yang WS (2012). Ferroptosis: an iron-dependent form of nonapoptotic cell death. Cell.

[9] Tenhunen R, Marver HS, Schmid R (1968). The enzymatic conversion of heme to bilirubin by microsomal heme oxygenase. Proc Natl Acad Sci USA.

[10] Tenhunen R, Marver H, Schmid R (1969). Microsomal heme oxygenase, characterization of the enzyme. J Biol Chem.

[11] Ryter SW, Choi AM (2009). Heme oxygenase-1/carbon monoxide: from metabolism to molecular therapy. Am J Respir Cell Mol Biol.

[12] Ferrándiz ML, Devesa I (2008). Inducers of heme oxygenase-1. Curr Pharm Des.

[13] Otterbein LE, Soares MP, Yamashita K, Bach FH (2003). Heme oxygenase-1: unleashing the protective properties of heme. Trends Immunol.

[14] Gueron G, Giudice J, Valacco P, Paez A, Elguero B, Toscani M, Jaworski F, Leskow FC, Cotignola J, Marti M, Binaghi M, Navone N, Vazquez E (2014). Heme-oxygenase-1 implications in cell morphology and the adhesive behavior of prostate cancer cells. Oncotarget.

[15] Andrés NC, Fermento ME, Gandini NA, Romero AL, Ferro A, Donna LG, Curino AC, Facchinetti MM (2014). Heme oxygenase-1 has antitumoral effects in colorectal cancer: Involvement of p53. Exp Mol Pathol.

[16] Yin H, Fang J, Liao L, Maeda H, Su Q (2014). Upregulation of heme oxygenase-1 in colorectal cancer patients with increased circulation carbon monoxide levels, potentially affects chemotherapeutic sensitivity. BMC Cancer.

[17] Li C, Lönn ME, Xu X, Maghzal GJ, Frazer DM, Thomas SR, Halliwell B, Richardson DR, Anderson GJ, Stocker R (2012). Sustained expression of heme oxygenase-1 alters iron homeostasis in nonerythroid cells. Free Radic Biol Med.

[18] Kadir FH, al-Massad FK, Moore GR (1992). Haem binding to horse spleen ferritin and its effect on the rate of iron release. Biochem J.

[19] Skouta R, Hayano M, Shimada K, Stockwell BR (2012). Dsign and synthesis of Pictet-Spengler condensation products that exhibit oncogenic-RAS synthetic lethality and induce non-apoptotic cell death. Bioorg Med Chem Lett.

[20] Immenschuh S, Baumgart-Vogt E, Mueller S (2010). Heme oxygenase-1 and iron in liver inflammation: a complex alliance. Curr Drug Targets.

[21] Lanceta L, Li C, Choi AM, Eaton JW (2013). Haem oxygenase-1 overexpression alters intracellular iron distribution. Biochem J.

[22] Weïwer M, Bittker JA, Lewis TA, Shimada K, Yang WS, MacPherson L, Dandapani S, Palmer M, Stockwell BR, Schreiber SL, Munoz B (2012). Development of small-molecule probes that selectively kill cells induced to express mutant RAS. Bioorg Med Chem Lett.

[23] Christofferson DE, Yuan J (2010). Cyclophilin A release as a biomarker of necrotic cell death. Cell Death Differ.

[24] Christofferson DE, Yuan J (2010). Necroptosis as an alternative form of programmed cell death. Curr Opin Cell Biol.

[25] Perrella MA, Yet SF (2003). Role of heme oxygenase-1 in cardiovascular function. Curr Pharm Des.

[26] Nishimura K, Tokida M, Katsuyama H, Nakagawa H, Matsuo S (2014). The effect of hemin-induced oxidative stress on erythropoietin production in HepG2 cells. Cell Biol Int.

[27] Lin Y, Zhang W, Qi F, Cui W, Xie Y, Shen W (2014). Hydrogen-rich water regulates cucumber adventitious root development in a heme oxygenase-1/carbon monoxide-dependent manner. J Plant Physiol.

[28] Li H, Wood JT, Whitten KM, Vadivel SK, Seng S, Makriyannis A, Avraham HK (2013). Inhibition of fatty acid amide hydrolase activates Nrf2 signalling and induces heme oxygenase 1 transcription in breast cancer cells. Br J Pharmacol.

[29] Sahin K, Tuzcu M, Gencoglu H, Dogukan A, Timurkan M, Sahin N, Aslan A, Kucuk O (2010). Epigallocatechin-3-gallate activates Nrf2/HO-1 signaling pathway in cisplatin-induced nephrotoxicity in rats. Life Sci.

[30] Sasaki T, Yoshida K, Kondo H, Ohmori H, Kuniyasu H (2005). Heme oxygenase-1 accelerates protumoral effects of nitric oxide in cancer cells. Virchows Arch.

